# Microglial cells: Sensors for neuronal activity and microbiota-derived molecules

**DOI:** 10.3389/fimmu.2022.1011129

**Published:** 2022-11-08

**Authors:** Giuseppina D’Alessandro, Francesco Marrocco, Cristina Limatola

**Affiliations:** ^1^ Department of Physiology and Pharmacology, Laboratory affiliated to Pasteur Italy, University of Rome La Sapienza, Rome, Italy; ^2^ IRCCS Neuromed, Pozzilli (IS), Italy

**Keywords:** microglia, gut microbiota, gut-derived molecules, antibiotics, gut brain axis

## Abstract

Microglial cells play pleiotropic homeostatic activities in the brain, during development and in adulthood. Microglia regulate synaptic activity and maturation, and continuously patrol brain parenchyma monitoring for and reacting to eventual alterations or damages. In the last two decades microglia were given a central role as an indicator to monitor the inflammatory state of brain parenchyma. However, the recent introduction of single cell scRNA analyses in several studies on the functional role of microglia, revealed a not-negligible spatio-temporal heterogeneity of microglial cell populations in the brain, both during healthy and in pathological conditions. Furthermore, the recent advances in the knowledge of the mechanisms involved in the modulation of cerebral activity induced by gut microbe-derived molecules open new perspectives for deciphering the role of microglial cells as possible mediators of these interactions. The aim of this review is to summarize the most recent studies correlating gut-derived molecules and vagal stimulation, as well as dysbiotic events, to alteration of brain functioning, and the contribution of microglial cells.

## Introduction

Microglia are multitasking cells that naturally respond to pathogens and maintain central nervous system (CNS) tissue integrity and functionality throughout life (1, 2). In the last decade, many studies deeper investigated on microglia phenotyping and functioning with new straightforward techniques in healthy and diseased brain (3, 4). In parallel, an increasing number of new data displayed the ability of gut microbiota in modulating microglia functions in healthy as well diseased conditions. Intrinsic and extrinsic factors such as host genetics, diet, lifestyle and drug use can significantly impact the gut microbiota, shaping both the microbial community and the pools of microbiota-derived metabolites (5–8). Here, we firstly summarize relevant studies on microglia identity and functions; then, we collected information on how microbiota-derived signals such as short chain fatty acids (SCFA), lipopolysaccharide (LPS), tryptophan derived molecules, vagal nerve stimulation and microbiota alteration affect the microglial phenotype and functions during steady-state and pathological conditions.

## Microglia: The brain resident macrophages

### Microglia control cerebral homeostasis

Microglia are the resident macrophages of the CNS that contribute to the innate immune surveillance of the brain and its homeostasis (9–11). Microglial cells derive from erythro-myeloid yolk sac precursors and before the maturation of the blood-brain barrier (BBB), they colonize the mouse brain at embryonic day 9.5 (12). Here, these cells proliferate and distribute throughout the CNS becoming the only myeloid cells in the healthy brain parenchyma (13); other myeloid cells, derived from the bone marrow, reside in the peri-parenchymal regions (perivascular and meningeal spaces) (14), or enter the brain only in pathological conditions (15). Microglia account for about 10% of brain cells, with some regional differences (16–18) and, together with astrocytes and oligodendrocytes, contribute to the non-neuronal part of the brain that supports the CNS environment (19). Microglial cells can be identified by specific markers, such as Transmembrane Protein 119 (TMEM119), Purinergic Receptor P2Y12 (P2RY12), and the Spalt-like transcription factor (SALL1) (20), however many of them are shared with macrophages (3). Alteration of certain markers such as the triggering receptor expressed on myeloid cells 2 (TREM2) and the Colony Stimulating Factor-1 receptor (CSF-1R) correlates with neurodegenerative diseases (21) and leukodystrophy (22) respectively indicating the important role played by these cells in the homeostatic control of CNS. Microglia continuously patrol cerebral parenchyma through their cellular processes and quickly react to pathological signals derived from acute and chronic injuries, neurodegenerative processes, or physiological aging (9, 11, 23). Microglial response to insults is often referred to as “activation” and includes migration or process extension to the damaged site, cell proliferation, phagocytosis, and production of soluble molecules (19); however, it must be mentioned that “activation” does not identify unique microglial phenotypes or functions. In particular, microglia may assume an ameboid-like or highly ramified shape upon different stimuli, increase or reduce its phagocytic activity and produce a number of cytokines, chemokines, and growth factors that may affect other glial or neuronal cell activities.

### Microglia secrete trophic and repair factors

According to the homeostatic role of microglia, these cells are able to secrete molecules that participate in the immune surveillance of the brain and produce neurotrophic factors important to neuronal homeostasis. In particular, microglial brain-derived neurotrophic factor (BDNF), is one of the main factors that regulates synaptic plasticity and spines density (24–26). Other evidence supports the involvement of microglia in the maturation and functioning of other brain cells. For instance, microglia-conditioned media pursue the differentiation program of neural stem/precursor cells (NSPCs) and oligodendrocyte precursor cells (OPCs) into astrocytes (27) and mature oligodendrocyte (28), respectively. Among the roles played by microglial cells to maintain cerebral homeostasis, tissue repair is one of the most prominent. As mentioned above, microglia constantly monitor the cerebral parenchyma, controlling neurons and their activity, performing a rapid response against intruders, and eliminating debris present throughout the cerebral environment. However, after injury or neural damage, microglia are also involved in tissue repair, through the release of molecules such as BDNF, tumor necrosis factor-alfa (TNFα), and Arginase-1 (29) that promote recovery from damage. Myelination is one important aspect of the recovery processes that involve microglia; this process requires iron, an important co-factor for oligodendrocytes (30), and microglia are the principal suppliers of this metal. Microglia also affect the re-myelination of damaged neurons with the release of molecules that promote the proliferation and differentiation of oligodendrocyte progenitor cells (OPC) (31). Re-myelination is a regenerative process that occurs even upon diseases such as multiple sclerosis (MS) but often fails in the progressive phase of the pathology (32). Tissue repair is a process that occurs after a natural immune response; however, an excessive phagocytic activity could damage tissues (33). To understand the role of activated microglial cells in tissue repair after an injury, Cunha and colleagues performed experiments in zebrafish and mice deleted from myeloid differentiation primary response gene 88 (MyD88), an adapter protein involved in cell-mediated immune response. In particular, they highlighted the importance of the pro-inflammatory phenotype in microglial cells to induce degradation and clearance of phagocytic myelin and to increase oligodendrogenesis (34). It has been widely accepted that regenerative properties are linked to the activity of immune cells; this is due initially to the activation of the pro-inflammatory phenotype that increases the proliferation of OPCs and, later, during the late phase of the immune response, microglial cells acquire an anti-inflammatory phenotype that releases growth factors that promote OPC differentiation (35). These results show the functions of microglia, from development to adult life, indicating not only the immune activity performed by these cells but also their roles in the maintenance of cerebral homeostasis

### Microglia promote neural circuits development and function

Another function performed by microglia in the CNS is to control the formation of neuronal circuits. In particular, brain circuits need microglia for proper development, functioning and maintenance of their plasticity. Microglia cells, similarly to macrophages, exhibit phagocytic activity, a receptor-mediated process that recognizes, engulfs and eliminates dead cells, bacteria or cellular debris. Among the receptors involved in this process are the toll-like receptors (TLR) that recognize microbial pathogens and TREM2, activated against apoptotic cells (36). During development, neuronal cells move to reach their final destination, but a high number of newborn cells will be eliminated before, during, and after the journey has been completed. This elimination mainly results from mechanisms of programmed cell death (PCD) (37). *In vivo* experiments indicated that microglia play an active role in this process and that the alteration of microglial activity deeply influences the elimination of neural precursor cells (NPCs) (38). It has been demonstrated that mice lacking the fractalkine receptor, C-X3-C Motif Chemokine Receptor 1 (CX3CR1), highly expressed on microglial cells, showed an increase in the number of apoptotic neurons in layer V of the cerebral cortex (39); this effect can be related to the activity of insulin-like growth factor 1 (IGF-1) a trophic factor implicated in NPC survival which is reduced in CX3CR1-deficient mice (40, 41). The connection between microglia and neural circuits, in addition to the normal building of the brain network, sees these cells also involved in the formation of memory. Synaptic plasticity is the neuronal event involved in long-term potentiation (LTP), one of the molecular mechanism that better explain the processes of learning and memory (42). Elimination of microglia cells with clodronate affects LTP in the hippocampal CA1 area, highlighting the role of these cells in the organization and preparation of circuits for memory formation (43). This key role is also observed during development, when mice lacking CX3CR1 show a reduction in both excitatory postsynaptic currents and glutamate release (44). Another important aspect of microglia in memory formation concerns the consolidation of the engrams (45). The role of microglia in the maintenance of memories is due to phagocytic activity and trogocytosis (46) that eliminates synapses (47). Eliminating the number of microglial cells with CSF-1R inhibitors, or reducing their phagocytic activity improves long-term effects on memory consolidation in mice (45). According to these data, microglia may play important roles in modulating neuronal elimination, The elimination of debris is important for correct neuronal networking: blockade of microglial CSF-1R affects their removal and neuronal connectivity (48). Moreover, the correct formation of the mouse visual system requires microglial activity for the pruning, which consists of the elimination of weaker or excessive synaptic connections, through the activation of the CR3 complement receptor in the retinogeniculate system (49). The pruning activity in this brain region is not mediated by CX3CR1 (50), which, however, contributes to the pruning of the barrel cortex and hippocampus (51, 52), indicating a spatial functional heterogeneity of microglia for the same activity. The application of high-throughput approaches for the study of single-cell RNA-seq led to the identification of microglial sensoma (53), common to a number of degenerative neurological conditions, but also to the discovery of spatially and temporally different microglial clusters that opens new perspectives for a more precise understanding of microglial roles in the brain (54).

### Identity and spatial heterogeneity

Microglial cells are characterized by dynamic changes from the early development until adult life, transferring from the yolk sac and then migrating into the CNS (12), where they proliferate and distribute to different areas (55); This activity highlights the presence of intrinsic changes in microglia both at the transcriptomic and functional levels, which ensure rapid adaptation to the environment. These changes can not only be related to the state of rest or activated microglia but highlight the presence of time- and space-dependent molecular programs. Recently, the use of advanced tools to study the gene expression programs of cells allowed to highlight the differences induced by space and time on myeloid cells (56). Matcovitch-Natan and colleagues, using RNA sequencing (RNA-seq) studied microglia gene expression from the embryonic stage until the adult brain (57). They described three microglial stages: the first, where microglia express genes related to the cell cycle such as minichromosome maintenance complex component 5 (Mcm5), and Disabled-2 (Dab2), the second, of pre-microglia, typical of the last embryonal stage, characterized by genes related to neuronal development such as Csf1, C-X-C Motif Chemokine Receptor 2 (Cxcr2) and, the last adult stage where microglia increase the expression of genes such as Cluster of Differentiation 14 (Cd14), Prostate transmembrane protein, androgen induced 1 (Pmepa1) (57). Recent work further improved this RNA-seq analysis, grouping the transcriptomic subpopulation of microglial cells through an independent component analysis (58). With this type of analysis Hammond and colleagues identified nine different microglial clusters in mice, with some genes predominant at specific ages, others common to all stages (Complement C1q A chain, C1qa; FC receptor-like S, Fcrls; Trem2) and others only transiently expressed (59). During the first stages of development, they observed the greatest variety of gene expression, which is reduced with time, becoming similar in the juvenile and adult phases. A similar description of microglial gene expression at the single cell level was obtained by Masuda (54). The spatial heterogeneity of microglia in different brain regions is not new (60); however, in Masuda’s work, this type of investigation has been extended to microglia under both physiological and pathological conditions. The homeostatic microglia have been classified into ten clusters segregated into two main groups belonging to the embryonic (C1-C6) and postnatal (C7-C10) stages. In the embryonic stage, these microglia groups are segregated in the embryonic CNS, as well as after birth where clusters are divided both spatially and temporally. In particular, in young mice, the C10 cluster of microglia was present in both the cortical region and in the hippocampus, whereas the C7 cluster was predominantly present in the cerebellum and corpus callosum of the adult (54). These data acquire a particular interest because they describe the different subtypes of microglia not only during the developmental phase but also in adult life, where the spatial distribution plays an important role in the characterization of microglia subpopulations. Interestingly, after an injury, specific microglia subtypes were recruited after three (C11) and fourteen (C12-C13) days, again indicating the presence of time-dependent phenotypes (54). Although they found several transcriptional models of gene expression in microglia, it is difficult to say whether these changes are related to different clusters, or rather they could represent a microglial adaptation to the environment. Microglia clustering is not a prerogative of the brain, but it has been also described in the spinal cord. This was confirmed by Tansley and colleagues by single-cells RNA sequencing (scRNA-seq) in both male and female mice in a nerve injury model. Interestingly, they confirmed the existence of several subpopulations of microglia, mostly belonging to six clusters; however, the situation changes after nerve lesions in different sexes. Male mice were characterized by a large transcription of pro-inflammatory genes, but the same intensity was not found in females, indicating an intrinsic difference between the two sexes to painful reactions. However, despite the different initial pro-inflammatory intensity, the immune response, after the injury, followed the natural course towards an anti-inflammatory transcriptional program (61). In addition, to confirm a different transcription program between the two sexes, they found an activated immune cluster (C9) only in male mice and, in females, a less intense proliferation program (61). Collectively, these papers, summarized in **Table 1**, have highlighted the existence of different microglial subpopulations that share common transcriptional programs, but that can quickly change based on different temporal and spatial signals from the environment or by the host.

**Table 1:** Microglia identity and spatial heterogeneity: Recapitulation of expressed markers and functions of clustered microglia in different temporal and spatial conditions.

(Hammond et al.,2019)		Uniquely expressed genes	Upregulated genes	Functions
*Youngest ages* Microglia (E14.5- P4/5)	C1	Trem2; C1qa; Fcrls	Arginase 1 (Arg1)
	C2a		Ribonucleotide reductase M2 (Rem2)	
	C2b		Ubiquitin-conjugating enzyme E2C (Ube2c)	
	C2c		Centromere protein A (Cenpa)	
	C3		Fatty acid binding protein 5 (Fabp5)	Participate to cell growth, motility, inflammation, and immunomodulation
	C4		Osteopontin (Spp1)	One major microglia state- participate to Axon Tract-Associated Microglia
	C5		Heme oxygenase 1 (Hmox1)	
	C6		Membrane-spanning4-domains, subfamily A, member 7 (Ms4a7)	Transmenbrane chemosensors that participate to immune cell function
Juvenile (P30) and Adult (P100) Microglia	C7a		Not defined by the expression of unique genes	
	C7b	
	C7c	
Adult (P100) and youngest (P5) Microglia	C8		Chemokine (C-C motif) ligand 4 (Ccl4)	
White matter Injury Microglia	C9		Interferon, alpha-inducibile protein 27 like protein 2A (Ifi27l2a)	
**(Matcovitch-Natan et al., 2016)**		**Upregulated genes**		**Functions**
Yolk sac	Y1	Lyz2; Pf4; Mcm5; Dab2; F13a1; Ifit3		Cell cycle and brain development
Early Microglia (E10.5-14)	E1	Dab2; Mcm5; Cdk1; Mbd2; Tpi1	
	E2	Hdac2; Dnmt1; Rad21;
Pre-Microglia (E14-P9)	P1	Cxcr2; Scd2; Psat1		Synaptic Pruning
	P2	Fcrls; Crybb1; Csf1
Adult Microglia (4 weeks-onward)	A1	P2ry13; Cx3cr1; Csf1r; Sall1		Immune surveillance
	A2	Selplg; MafB; Pmepa1; CD14
**(Masuda et al.; 2019)**		**Upregulated genes**		**Functions**
Embryonic microglia	C1	Ctsb; Ctsd; Lamp1; Apoe	
	C2	Ctsb; Ctsd; Lamp1
	C3	
	C4	Apoe
	C5	Apoe	
	C6	Tmsb4x; Eef1a1; Rpl4;
Post-natal Microglia	C7	Tmem119; Selplg; Slc2a5		Microglia Homeostatic genes
	C8	Tmem119; Selplg; Slc2a5
	C9	Tmem119; Selplg; Slc2a5; Cst3; Sparc
	C10	Tmem119; Selplg; Slc2a5; Cst3; Sparc
Microglia After demyelination and unilateral facial nerve axotomy, (FNX)	C11	Ctsc;		
	C12	Fam20c; Cst7; Ccl6; Fn1; Ank; Psat1; Spp1
	C13	Cybb; Cd74; H2-Aa; H2-Ab1 amd MHC class II genes

The letter in the table are the name of the clusers defined by each author in each paper revisioned. As described in a note next to the table, a column titled clusters is needed in the table.

## Microglia functions in the brain

### Microglia in healthy brain

Microglia represent the resident myeloid cells that inhabit the cerebral parenchyma and contribute to its surveillance and maintenance. Among the homeostatic functions of microglial cells, their neuromodulatory properties have been deeply documented (62). These activities are made possible by the presence of several receptors on microglial cells, among them, purinergic receptors which recognize adenosine triphosphate (ATP) and their metabolites, signals commonly used by neurons to communicate with microglia (63). Bidirectional communication between microglial cells and neurons is also important to stimulate the movement of microglial cells towards neurons to modulate their activity (63). Recently, ATP-dependent microglia activation has been correlated to an inhibitory program to reduce excessive/dangerous neuronal activity (64). This stimulation of purinergic receptors increases the release of BDNF, which plays an important role in brain neuromodulation; in particular, this neurotrophic factor is implicated in both neuronal differentiation and synaptic plasticity (65); specific depletion of BDNF from microglia does not modify its brain levels but reduced the formation of new synapses in the cerebral motor cortex and the motor learning abilities in mice (66). Among the physiological activities of microglia, it has been recently shown that these cells participate in the regulation of sleep, in mice. It has been described that the accumulation of ATP during the sleep phase reduces the microglial expression of CX3CR1 and that microglial depletion increases the duration of NREM sleep during the wake phase, with a simultaneous reduction of the hippocampal excitatory neurotransmission in mice (67). Altogether, these data indicate that, under physiological conditions, microglia participate both in the homeostatic balance and synaptic activity of the brain microenvironment.

### Microglia in brain diseases

Considering the fundamental role of microglial cells in the brain, it is not surprising that they also participate in brain disorders, as well as inflammatory diseases of the brain. Alzheimer’s disease (AD) is a progressive neurodegenerative disorder characterized by general cognitive impairment and with two distinctive pathological signs: extracellular plaques composed of amyloid β-peptide (Aβ) and intraneuronal tangles composed of hyperphosphorylated tau proteins. Both patients and animal models of this pathology are characterized by microglial cells with altered phagocytic activity around the amyloid plaques (68). In animal models of AD, extensive genome-wide association studies (GWAS) have identified many immune-related genes associated with high-risk factors for this pathology, which are highly expressed in microglial cells (48). Among these genes, mutated forms of TREM2 represent a pivotal risk factor for this neurodegenerative disease (69). TREM2 controls the activity of microglial in synapses, and the elimination or mutations on this receptor contribute to the deposition of amyloid plaques in mice brains (70) as well as to an energy imbalance that increases microglial cell dysfunction (71). These data, in agreement with others, attribute to microglial cells an active role in fighting AD since it is possible that wrapping the amyloid plaques helps to form a protective barrier that reduces the neurodegeneration process progression. In different neurodegenerative diseases, such as Parkinson’s disease characterized by motor impairment, microglia behave differently, contributing to neuroinflammation and speeding up neurodegeneration. For instance, mutations in leucine-rich repeat kinase 2 (LRRK2) are associated with different forms of PD (monogenic and sporadic) in an age-dependent manner (72). LRRK2 is a serine/threonine kinase (73), and mutations in this protein increase kinase activity and contribute to neurodegeneration in PD (74). LRRK2 increases the pro-inflammatory phenotype in α-synuclein-treated microglia (75), whereas in LRRK2-deficient rats the inflammatory phenotype mediated by this fibrillar aggregate is reduced (76). Even if the *in vivo* effects of this mutated protein on microglial cells are controversial, the relationship between inflammation and LRRK2 on resident brain macrophages is defined. In particular, exposure of microglia to LPS increases the inflammatory phenotype and the level of LRRK2, while inhibition of LRRK2 reduces the production of pro-inflammatory cytokines (77). In addition, LRRK2 appears to regulate microglial motility, reducing its migration (78) and favoring the local inflammation state. Together, these data strengthen the knowledge of the role of LRR2 in neurodegeneration and the effect of microglial cells as operators of the inflammatory state observed in PD. Among diseases affecting the brain, synchronized hyperactivation of neural cells causes epilepsy, a neuropsychiatric disorder that occurs when excitatory activation of neurons exceeds the inhibitory (79). Changes in microglia were also observed after seizures: in the hippocampus of mice treated with kainic acid, microglial cells showed an activation that persists even 24 hours after injections as a side effect of hyperactivity (80, 81). Other evidence support the contribution of microglial cells to brain hyperactivity due to inflammatory response. In particular, the release of cytokines stimulates neuronal activity, favoring epileptogenesis (82) The activation of TLRs, which promote microglial activity, affects seizures: mice lacking the TLR3 or mice treated with TLR4 antagonist show an attenuation of recurrent spontaneous seizures after injections of pilocarpine (83) and kainate (84), respectively. Neuroinflammation is a common condition in brain diseases, and since microglia are the main cells that direct inflammation in the brain, it is not surprising that they are involved in many neural disorders. Among mental illnesses, depression is a condition that affects people and causes emotional suffering; the events that can generate the depressive state are many, however, a common condition is the inflammatory state of the brain. Interferon-gamma (IFN-γ) is a cytokine that promotes microglial activation: Zhang and colleagues demonstrated that intracerebroventricular injection of IFN-γ impaired hippocampal neurogenesis and induced depressive-like behaviors in mice, effects reduced using minocycline to inhibit microglia (85).The works described until now, clearly demonstrated that microglia have distinct roles in a healthy brain, organizing the functions to maintain homeostatic balance; however, there are clear proofs supporting the contribution of microglial cells even in brain disease, when these cells can improve or worsen disease progression. Together, these evidence suggest that microglial cells could represent a promising therapeutic target for brain diseases.

## Gut microbiota-derived molecules controlling microglia in healthy and diseased brain

**Figure 1:**
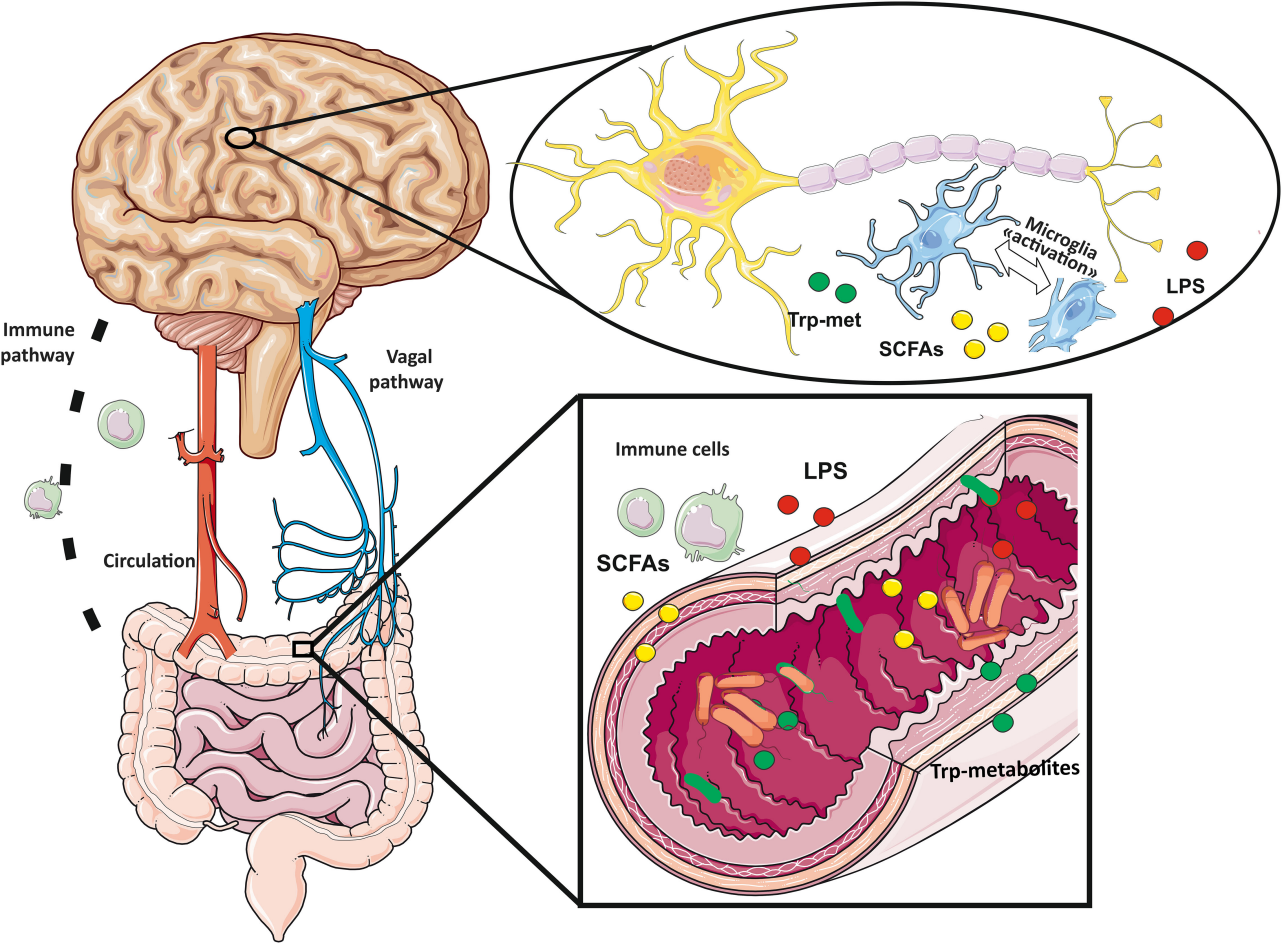
Gut-brain axis follows bidirectional communication routes: vagal nerve, immune system pathways and circulating small molecules produced by bacteria (tryptophan derived metabolites, short chain fatty acids and lipopolysaccharides) reaching the brain through the circulation.

Microbiota is the term used to describe all the microorganisms (bacteria, yeasts, archaea, protozoa and viruses) living on the epithelial barriers of the human body (nose, eye, skin, lungs, gastrointestinal, genital and urinary tract). The human gut is the natural habitat for more than 100 trillion of microorganisms. The intestinal microbiome, i.e. the totality of the genetic heritage owned by the intestinal microbiota, encodes over three millions of genes and produces thousands of molecules important for host health. These molecules exert both local and peripheral effects maintaining the homeostasis of intestine and distant organs. Small molecules, such as the short chain fatty acids (SCFAs), lipopolysaccharide (LPS) or tryptophan (trp) metabolites are produced in the colon and largely absorbed by the colonocytes; part of them reach the systemic circulation, playing key roles in microbiota-gut-brain cross-talk (**Figure 1**). A second route for gut-brain communication is represented by signal through the vagal nerve. It ensures bidirectional communication, using both neurotransmitters such as serotonin and glutamate and, gut hormones (**Figure 1**). Healthy gut microbiota is fundamental for maintaining normal host functioning; indeed, alteration of the gut microbiota and its metabolites, defined as gut dysbiosis, can induce harmful effects on the host. The imbalance in microbiota composition leads to a wide range of inflammatory and metabolic pathologies including brain diseases (86–89). In the following paragraphs the most studied gut microbiota-derived molecules, vagal nerve stimulation and their effects on microglia will be reviewed.

### SCFAs and microglia

The fermentation of fibers contained in the food by colon microbes produces bioactive molecules. Among them, the short-chain fatty acids (SCFAs; e.g., formate, valerate, acetate, propionate, and butyrate) which are small molecules ranging from 1 to 4 atoms of carbon. It has been shown that SCFAs can act through either G-protein-coupled receptors (GPCRs) or histone deacetylases (HDACs) (90, 91).

One of the first evidence showing the role of microbiota-derived molecules on microglia was obtained in germ-free (GF) mice by Erny and colleagues in 2015. The authors showed that GF mice displayed global defects in microglia, with altered cell shape and immature phenotype, leading to impaired innate immune responses. Classical SCFAs supplementation to GF mice reverted microglial phenotype and lipopolysaccharide response (92).

Later, the same authors identified microbiota-derived acetate as critical driver of microglia maturation and regulator of the homeostatic metabolic state in germ free mice. Further, they also show the ability of acetate to modulate microglial phagocytosis and disease progression in one murine model of Alzheimer’s disease (93). Recently, it has been observed that SCFAs can reverse the age-related pro inflammatory state of microglia. In aged mice, the supplementation of inulin, a prebiotic fermentable fiber, increased the endogenous levels of SCFAs and reduced the expression of many inflammatory genes, as demonstrated by scRNA-seq on microglial cells. In addition, microglia from aged mice spontaneously secreted more TNF-α than microglia from adults and this effect was reduced in microglia of aged mice fed with inulin (94). On the other hand, a fiber-deprived diet altered gut microbiome, reducing SCFAs and inducing cognitive impairment in mice. In particular, dietary fiber deficiency for 15 weeks correlated with microglia-mediated synaptic loss in the hippocampus, suggesting that fiber intake could represent a nutritional preventive strategy to reduce the risk of cognitive decline and neurodegenerative disease (95). In mouse models of Alzheimer’s disease, the expression of the synapse-associated proteins (Postsynaptic Density protein- 95-PSD-95; Synaptophisyn-SYP; N-methyl-D-aspartate receptor 2 B -NR2B) are reduced and the pro-inflammatory cytokines (TNF-α, IL-6, IL-1β) are increased. These alterations could be reversed treating mice daily, for 2 weeks, at early stage of disease, with sodium butyrate by intraperitoneal injection. In particular, SCFA treatment suppressed the over-activation of microglia and the accumulation of Aβ in AD mice (96). It has been shown by Sadler and colleagues that stroke alters the gut microbiota composition, inducing dysbiosis and a plasma decrease of SCFAs. The authors showed that classical SCFAs supplementation in drinking water for 4 weeks improved the behavioral outcome after stroke, reducing brain microgliosis and the expression of the phagocytosis marker CD68 in the ipsilateral hemispheric cortex (97). Housing rodents in an enriched environment (EE), a condition characterized by abundant social interactions, and cognitive, sensory, and motor stimulations could improve their learning and memory abilities (98). Recently, it has been shown that housing mice in an EE affects their gut microbiota and metabolome composition, elevating the concentration of specific SCFAs, with effects on synaptic plasticity processes (99). In the same year, Lupori and colleagues showed that classical SCFAs (acetate, propionate and butyrate) treatment changed microglia morphology in the visual cortex toward a hyper-ramified condition characteristic of high plasticity, similar to EE, favoring ocular dominance plasticity process (100). On the other hand, in a model of neuropathic pain induced by chronic constriction injury (CCI), Zhou and colleagues observed signs of mechanical and thermal pain, and an increased expressions of microglia (Iba1, CD11b) and pro-inflammatory markers (CD68, IL-1β, and TNF-α) both in the hippocampus and the spinal cord. These phenomena were accompanied by increased level of SCFAs in the gut. Antibiotic treatment reversed microglia activation but its action was blocked by SCFA administration, suggesting these molecules as key player in the pathogenesis of neuropathic pain (101). Recently, in α-synuclein overexpression and MPTP (1-methyl-4-phenyl-1,2,3,6-tetrahydropyridine) models of Parkinson’s disease (PD), the oral supplementation with SCFAs (mixture of acetate, propionate and butyrate or butyrate alone) worsened the disease progression, increasing microglia activation and TNF-α release (102, 103). Further researches are needed to clearly define the role of SCFAs, considering both the beneficial and detrimental effects described above on microglia and neuroinflammatory diseases.

As reviewed above, authors mainly described the effect of SCFAs mixture *in vivo*. In contrast, Wenzel and colleagues described the effect of single or combined treatment on human microglia cell line THP-1 with acetate, propionate, butyrate, formate, and valerate at an approximate physiological concentration ratio. They found that the SCFA mixture, as well as single SCFAs (at the highest concentrations used in the mixture, i.e. from 15 to 236 μM), decreased the secretion of IL-1β, MCP-1, TNF-α, and cytotoxins induced by LPS stimulation. In addition, formate and valerate reduced the phagocytic activity of LPS-stimulated THP-1 cells. Formate, but not valerate, also inhibited the N-formylmethionine-leucyl-phenylalanine (fMLP)-induced respiratory burst of HL-60 cells, reducing the production of reactive oxygen species (ROS) (104).

Altogether, these researches identify SCFAs as the key soluble links along the gut-brain axis and propose them as potential therapeutic tools to target microglia-related brain diseases.

### LPS and microglia

Changes in the gut microbiota leading to the expansion of gram-negative bacteria increase the release of microbial LPS, a bacteria cell wall component. LPS activates the TLRs, membrane-spanning receptors expressed in microglial cells, which recognize common damage- or pathogen-associated molecular-patterns (DAMPS, PAMPs) (105). LPS can stimulate host immunity and the immunological response depends on the origin of the microbial species. Indeed, the lipid A portion of LPS is variable and contains the endotoxic component, thus contributing to the structural and functional diversity of LPSs among microbial species (106). In this regard, an *in vitro* study showed that exposure of rat neonatal microglia to LPS from *Cyanobacterium Oscillatoria sp*. resulted in a concomitant release of proinflammatory and anti-inflammatory mediators (107). In contrast, a study that treated rat microglia with LPS from *Cyanobacterium Microcystis aeruginosa* described the induction of a classical (or M1-like) microglia state and the release of pro-inflammatory mediators (108).

It has been shown that stress decreases the level of Lactobacillus and Bifidobacterium and increases the gram-negative bacteria, with an increase of LPS (109, 110). Chronic LPS release activates systemic inflammation and neuroinflammation, both effects being crucial for the pathogenesis of neurodegenerative and psychiatric disorders (111). Indeed, elevated levels of LPS are observed in amyotrophic lateral sclerosis (112), AD (113) and severe autism (114).

Neuroinflammation, indeed, mainly comprises the over activation of microglia cells that can became harmful for brain parenchyma.

It has been shown that intraperitoneal injection of LPS increases the number of Iba-1 positive cells in different brain region, in a dose-dependent manner in conventional mice, but not in GF mice (115, 116) underlying the key role of microbiota in driving LPS-mediated effects.

As reviewed, several factors can alter the abundance of LPS in the host; however, the actual role of microbiota derived -LPS in activating microglia remains to be investigated.

### Tryptophan-derived ligands of aryl hydrocarbon receptor and microglia

In addition to SCFAs, it has been recently demonstrated that aryl hydrocarbon receptor (AhR) ligands secreted by gut bacteria can influence microglia. Tryptophanase-expressing bacteria catalyze the conversion of dietary tryptophan to indole, the precursor for the synthesis of AhR agonists (indoxyl-3-sulfate and indole-3-propionic acid). Rothhammer and colleagues showed that AhR activation by dietary tryptophan (Trp) metabolites suppressed the microglial expression of NF-κB dependent transcripts such as *Tnfa* and regulated microglial expression of *Tgfa* and *Vegfb* in a mouse model of multiple sclerosis, the experimental autoimmune encephalomyelitis (EAE) (117, 118). The same effects were shown in human microglia, activating AhR by the tryptophan metabolite indoxyl-3-sulfate (I3S). AhR activation suppressed the expression of pro-inflammatory and neurotoxic genes (*TNFA, IL6, IL12A, NOS2*) and boosted the expression of anti-inflammatory IL10; AhR activation also induced *TGFA* and reduced *VEGFB* expression in human microglia (118). The dietary metabolites and other AhR ligands, such as indirubin-3′-oxime, were also shown to inhibit the inflammatory phenotype of microglial cells in rat brain (119). On the other hand, in a model of mouse middle cerebral artery occlusion (MCAO), Tanaka and colleagues showed that AhR expression increased in microglia during ischemia. Pre-treatment with an AhR antagonist, CH223191, significantly reduced the expression of TNFα, IL-1β and cyclooxygenase-2 (COX-2) in the cortex and striatum of MCAO mice (120). Altogether, these data suggest that microbial tryptophan and other AhR ligands can modulate microglia, adding new therapeutic targets for brain diseases with inflammatory components.

### Quorum sensing peptides and microglia

Among the bacterial derived molecules able to modulate microglia, the quorum sensing peptides (QSPs) have been recently described. QSPs are oligopeptides produced by Gram-positive bacteria to communicate with their peers in a cell-density dependent manner. In particular, Janssens and colleagues described the ability of an active heptapeptide (SDLPFEH, named PapRIV) originating from members of Bacillus cereus group. The authors showed that PapRIV is present in mouse plasma and that can reach the brain, crossing the brain blood barrier (121). In addition, they described that PaPRIV has pro-inflammatory effects on the microglial BV-2 cells, inducing the expression of IL-6 and TNF-α, along with an increase of intracellular ROS. This is the first evidence for a possible role of this bacterial quorum sensing peptide in gut-to-brain signaling (121).

### Vagal nerve stimulation and microglia

Vagal afferent neurons located in the nodose ganglion (NG) innervate the gut and terminate in the nucleus of solitary tract (NST) of the brainstem. Vagal afferent terminals are located below the gut epithelium, where they sense signals derived from the gut microbiota, with effects on host behavior. The NST is a target for gastrointestinal signals modulating satiety, and alterations in the gut-brain vagal pathway may promote overeating and obesity. In rats treated with high fat diet (HFD), the alteration of microbiota composition triggered the reorganization of vagal afferents and the activation of microglial cells in the NST (122). Similar data were obtained in rats fed with low fat/high sugar diet (123). Kim and colleagues demonstrated that HFD-induced dysbiosis, *per se*, decreased vagal innervation and increased Iba1 positive cells in the NG of GF rats which received fecal transplantation from HFD- (45 or 60% fat) or low-fat diet- (LFD, 13% fat) treated rats (124). These studies suggested that HFD-triggered shift in gut microbiome may affect the vagal gut-brain communication resulting in microglia activation and increased body fat accumulation.

It has been reported that the electrical stimulation of the vagus nerve, in the presence of a peripheral immune challenge may affect microglial cells, up-regulating anti-inflammatory pathways in the brain (125, 126). Vagus nerve stimulation, combined with LPS challenge, decreases microglial production of the pro-inflammatory cytokines IL-6, IL-1β, and TNFα, an effect abolished upon vagotomy (127). These data suggested that vagus nerve activity reduces neuroinflammation. In line with this observation, in a rat model of transient ischemia, berberine, an alkaloid with weak antibiotic properties, decreased CD86-positive and increased CD163-positive microglial cells in the cerebral cortex, in a microbiota-dependent manner. The authors show that the hydrogen sulfide (H_2_S) derived from microbiota metabolization of berberine, stimulates the vagus nerve through the transient receptor potential vanilloid 1 (TRPV1) and that a cocktail of antibiotics blocked vagal activation, confirming the necessity of a functional metabolizing microbiota (128).

Recently, Yunpeng and colleagues showed that *Lactobacillus rhamnosus* (JB-1) has anxiolytic effects and decreases Iba1+ microglial cells in mouse hippocampus. The authors also demonstrated that the loss of vagal integrity, obtained by subdiaphragmatic vagotomy, inhibited these effects, further pointing to the important role of vagus nerve in the signaling between the gut microbiota and the brain (129). Although different studies performed in different models reported that microglial cells are modulated by vagal activation, the relationships between the vagus nerve and the immune signaling, in microbiota-gut-brain communication, need further investigation.

## Gut microbiota modulation by antibiotics in healthy and disease: The effects on microglia

The use of antibiotics (ABX) to eliminate or unbalance the gut microbiota is a useful tool to study the relationship of specific bacterial cohorts and different brain functions in adult animal models. At difference with the GF mice, which permit to study the role of the gut microbiota during development, ABX, especially the non-absorbable ones, open to the opportunity to study the role of gut microbes and their associated molecules in healthy mice or murine brain disease models.

In adult healthy mice, two-weeks of ABX treatment influence hippocampal microglial density, reducing their basal patrolling activity, and impairing process rearrangement in response to damages (130). In addition, ABX treatment induced a reduction of spontaneous postsynaptic glutamatergic currents and in general, of synaptic connectivity (110).

Interestingly, in adult healthy mice, it has been demonstrated (by RNA-seq of FACS-purified microglia) that one-week of ABX treatment triggered transcriptomic changes in microglia, in a sex dependent manner. In particular, Thion and colleagues showed that ABX treatment induced different expression of 92 microglial genes in male mice, and of only 40 genes in female mice, suggesting a sexually dimorphic impact of microbiota on microglial transcriptomes (131).

A more prolonged (4 weeks) ABX treatment, aimed to fully eradicate the gut microbiota, led to microglia activation, with an increase of CD40 and MHC II expression, IL-6 and TNF-α production, and more Iba1+ cells in the hippocampal CA3 and CA1 regions (112). Theis treatment also induced a reduction in the synaptic transmission measured as hippocampal cholinergic gamma power oscillations in the CA3, after ABX treatment (132).

Recently, many reports showed that the intestinal microbiota also influences the neurodevelopment, the behavior, and contributes to brain disorders. On the other hand, gut microbiota dysbiosis is observed in a number of brain disorders characterized by microglia dysfunction such as autism spectrum disorders (ASD), schizophrenia, AD, major depressive disorder, and PD (133–144)

In a murine model of Herpes simplex encephalitis (HSE), a complication of herpes simplex virus type I infection, Li and colleagues observed alterations in gut microbiota composition. In these mice, the use of oral ABX aggravates the virus-induced pathology, triggering the pro inflammatory activation of microglia. The administration of another microbial derived product, nicotinamide n-oxide, significantly diminished inflammation in ABX-treated or untreated HSE mice (145).

In a murine model of familial ALS (hSOD1^G93A^ mice), it has been shown that a combination of 8 antibiotics (146) worsened the motor functions and reduced survival, exacerbating the pro-inflammatory phenotype of microglial cells in the spinal cord. The authors found that ABX treatment reduced the relative abundance of *Akkermansia* and of butyrate-producing bacteria such as *Clostridium g24, Ruminococcus*, and genera in the *Lachnospiraceae* family, that are triggers of anti-inflammatory responses (147, 148). These data suggest that these microbes could play a beneficial role at least in the familial form of ALS (149). Similarly, in a murine model of AD (the APPS1 model) the administration of *Akkermansia muciniphila* by oral gavage, every day for 6 months resulted in a delay of the pathological changes in the brain and ameliorated the spatial learning and memory tests (150).

On the other hand, it has been shown that, in two independent transgenic mouse models of AD (APPSWE/PS1ΔE9 and APPPS1-21(APPSWE/PS1L166P mice), only males showed reduced Aβ amyloidosis and altered phenotypes of plaque-associated microglia following administration of an ABX cocktail. In particular, ABX treatment altered the levels of selected microglial transcripts for homeostatic proteins in male but not in female mice (151, 152). More recently, the same authors showed that CSF-1R inhibitor-mediated depletion of microglia in ABX-treated male mice failed to reduce cerebral Aβ amyloidosis. Thus, they demonstrated the key role of microglia in bridging gut microbiota-mediated modulation of cerebral Aβ deposition (153). In a different mouse model of AD (5 x Familial AD, 5xFAD), it has been recently shown that GF or ABX treated mice differentially control the microglial mechanisms of Aβ clearance, preventing both neurodegeneration and cognitive deficits. While both conditions attenuated hippocampal pathological signs, only GF 5xFAD mice enhanced microglial Aβ uptake at early stages compared to ABX-treated 5xFAD mice. These observations were supported by RNA-sequencing of hippocampal microglia from control, GF and ABX-treated 5xFAD mice, that showed distinct profiles of microbiota-dependent gene expression associated with phagocytosis and altered microglial activation states (154). Different genetics and ABX treatments lead to different outcomes in microglia activation in the context of the same pathology, suggesting a plastic role of gut microbiota-microglia axis in AD.

In the last decade, the importance of gut microbiota in multiple sclerosis (MS) has been highlighted: many studies reported the presence of gut microbiota dysbiosis in patients (155–157).

In a murine model of progressive MS triggered by intracranial infection with Theiler’s murine encephalomyelitis virus (TMEV), Mestre and colleagues treated mice with oral ABX in the pre-symptomatic and symptomatic phases of the disease. The cocktail of ABX prevented motor dysfunction and limited axon damage in mice. In the spinal cord of TMEV mice treated with ABX, microglia assume a round amoeboid morphology associated to an anti-inflammatory gene profile (increase of IL-4 and IL-10) (158). Similar results were obtained in a different murine model of MS where a pretreatment of either three or seven days with oral ABX cocktail (Ampicillin, Metronidazole, Neomycin Sulfate, and Vancomycin) protected mice from signs of EAE. In addition, RT-PCR data on spinal cord tissue showed an upregulation of *Arg1*, a gene associated with anti-inflammatory microglia/macrophages and a downregulation of the pro-inflammatory genes iNos and TNF-α (159).

ABX treatment resulted beneficial also in one murine model of PD with α-synuclein (αSyn) overexpression, showing motor dysfunction and gastrointestinal (GI) constipation. In these mice, Sampson and colleagues reported that ABX treatment resulted in milder αSyn-dependent motor dysfunction and an increased GI function. Microglia showed morphological changes that indicated an arrest in maturation and/or a reduced activation state, suggesting that postnatal signaling between the gut and the brain modulates the disease (102).

Recently, the effect of gut microbiota alteration was studied also in brain tumor. Treating mice with two non-absorbable ABX (gentamicin and vancomycin) for two weeks prior to glioma transplantation in the brain reduced the cytotoxic NK cell subsets and altered the expression of inflammatory and homeostatic proteins in microglia. All these effects could contribute to the increased growth of intracranial glioma observed in ABX-treated mice (160).

In one rat model of major depression, where animals show high anxiety-like behavior, lower microglial numbers in prefrontal cortex and altered gut microbiota composition, Schmidtner and colleagues demonstrated that three-week treatment with oral minocycline, a broad-spectrum tetracycline antibiotic, alleviated the depressive-like phenotype. In addition, this treatment further reduced prefrontal microglial density, exclusively in male rats, and reduced the plasma concentrations of pro-inflammatory cytokines. These results support the microbiome-gut-brain axis as potential target in the treatment of depression (161).

The modulation of gut microbiota by antibiotics revealed several unexpected gut-brain links in many pathologies, worth to be further elucidated. However, ABX treatment could be also considered a new therapeutic strategy to be used not only for infectious diseases, but also for brain disorders associated with gut microbiota dysbiosis. Finally, it must be considered that there are many other ways to modulate the microbiota and therefore the microglia, not discussed here. These include, for example, fecal microbiota transplantation, administration of pro/prebiotics (e.g. sodium oligomannate) and/or live biotherapeutics.

## Closing remarks

A number of recent studies demonstrated that the gut microbiota is a powerful modulator of microglial phenotype and functioning, in healthy and disease conditions. However, the molecular mechanisms used by the gut microbiota and the metabolome to impact host neuroimmune cells need further investigations. The availability of new DNA sequencing strategies, such as shotgun metagenomic sequencing and metabolomics will allow the analysis of gut microbiome composition at the level of species and will permit the identification of specific microbe cohorts and their cross-correlation with microbial metabolites in the different conditions. Therefore, we propose to investigate how the modulation of specific bacterial species or cohorts (by specifically depleting or enriching them) might result in microglia alteration in the brain of healthy and diseased murine models. Further, manipulation of microbiota composition by pre-biotics, environmental stimuli, dietary habits, and fecal transplantation might represent new therapeutic strategies to for the treatment of brain disorders involving microglia.

## Author contributions

CL, GD and FM contributed to the conception, design, and writing of the manuscript. FM and GD designed the figure. All authors have read and agreed to the published version of the manuscript. All authors contributed to the article and approved the submitted version.

## Funding

This work was supported by PRIN 2017, AIRC 2019 -IG 23010, RF-2018-12366215 to CL and by the European Union’s Horizon 2020 Research and Innovation Program under grant agreement No. 952455; EpiEpiNet (CL); GR-2016-02363254 to GD.

## Conflict of interest

The authors declare that the research was conducted in the absence of any commercial or financial relationships that could be construed as a potential conflict of interest.

## Publisher’s note

All claims expressed in this article are solely those of the authors and do not necessarily represent those of their affiliated organizations, or those of the publisher, the editors and the reviewers. Any product that may be evaluated in this article, or claim that may be made by its manufacturer, is not guaranteed or endorsed by the publisher.
